# Supplementary figures and images for: Acute Physiological Response to Different Sprint Training Protocols in Normobaric Hypoxia
Source: Int J Environ Res Public Health. 2022 Feb 24;19(5):2607. doi: 10.3390/ijerph19052607 (PMC8909614; doi:10.3390/ijerph19052607)

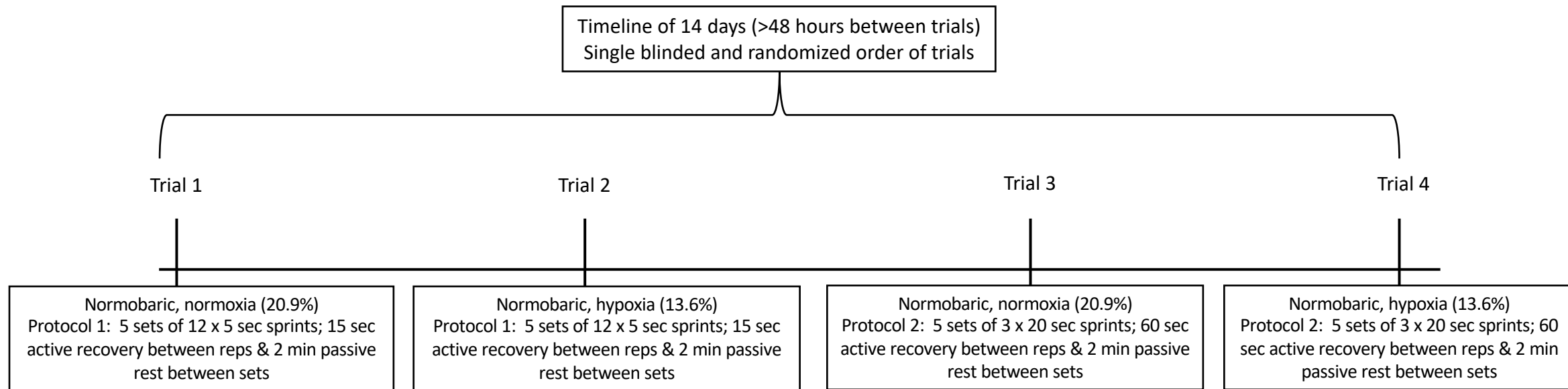

**Figure S1.** Study Timeline.

Supplement: Supplementary file 1 [file ijerph-19-02607-s001.zip › ijerph-1571460-supplementary.pdf]
